# Stereotactic Percutaneous Electrochemotherapy as a New Minimal Invasive Treatment Modality for Primary and Secondary Liver Malignancies

**DOI:** 10.3390/biomedicines12122870

**Published:** 2024-12-17

**Authors:** Lukas Luerken, Andrea Goetz, Vinzenz Mayr, Liang Zhang, Alexandra Schlitt, Michael Haimerl, Christian Stroszczynski, Hans-Jürgen Schlitt, Matthias Grube, Arne Kandulski, Ingo Einspieler

**Affiliations:** 1 Department of Radiology, University Hospital Regensburg, 93053 Regensburg, Germany; 2 Department of Diagnostic and Interventional Radiology, Klinikum Würzburg Mitte gGmbH, 97070 Würzburg, Germany; 3 Department of Surgery, University Hospital Regensburg, 93053 Regensburg, Germany; 4 Department of Internal Medicine III, Hematology and Oncology, University Hospital Regensburg, 93053 Regensburg, Germany; 5 Department of Internal Medicine I, Gastroenterology, Hepatology, Endocrinology, Rheumatology and Infectious Diseases, University Hospital Regensburg, 93053 Regensburg, Germany

**Keywords:** HCC, iCCA, liver cancer, liver metastasis, interventional oncology, percutaneous tumor ablation, electrochemotherapy, stereotactic navigation, CT-guided intervention

## Abstract

**Background and Objectives**: To report on the first results of safety, efficacy, and outcome of CT-navigated stereotactic percutaneous electrochemotherapy (SpECT) in patients with primary and secondary liver malignancies. **Methods**: This retrospective study included 23 consecutive lesions in 22 patients who underwent SpECT for primary and secondary malignant liver lesions with locally curative intention. The endpoints were primary technique efficacy (PTE), local tumor progression (LTP), time to progression (TTP), and occurrence of adverse events. **Results**: The mean maximum diameter of the treated lesions was 42 mm (range: 16 mm–72 mm). Eight lesions were hepatocellular carcinoma (34.8%), five lesions were colorectal liver metastases (21.7%), three lesions were cholangiocellular carcinoma (13.0%), and the other seven lesions were liver metastases from different primary cancers (30.4%). PTE was achieved for 22 lesions (95.7%). The mean follow-up time was 15 months (0–39 months). No LTP was observed. In six patients (27.3%), hepatic tumor progression was observed during follow-up with a mean TTP of 3.8 months (2–8 months). In 10 procedures (43.5%), minor complications (1 CIRSE Grade 2) and side effects occurred, but no major complications were observed. **Conclusions**: SpECT seems to be a safe and effective new local treatment modality for primary and secondary liver malignancies.

## 1. Introduction

Primary and secondary liver malignancies are common and represent a significant worldwide health concern. Primary liver malignancies, especially hepatocellular carcinoma (HCC) and, to a lesser extent, intrahepatic cholangiocarcinoma (iCCA), as well as other less common malignancies like hemangioblastoma, are amongst the leading causes of cancer-related deaths worldwide [1,2]. Secondary hepatic malignancies are a more heterogeneous group of diseases and are even more common than primary liver tumors [3], with colorectal cancer, lung cancer, breast cancer, and prostate cancer as the most frequent primary malignancies for liver metastases [2,4,5,6,7]. In many clinical scenarios, surgical resection is the therapy of choice for the treatment of primary liver tumors and oligometastatic secondary liver tumors. However, in many cases, surgical removal of liver tumors at the time of diagnosis is not possible [8,9].

Minimally invasive local ablative treatment approaches are increasingly being pushed into this gap, given the inclusion in several international guidelines for the multimodal treatment of primary and secondary liver malignancies [10,11,12,13]. The most established local ablative methods are hyperthermic percutaneous treatment options, including radiofrequency ablation (RFA) and microwave ablation (MWA). Despite the advantages of MWA and RFA compared to liver surgery regarding invasiveness, repeatability, and cost-effectiveness [14], limitations like the heat sink effect and the risk of thermal damage to thermosensitive intrahepatic structures like major bile ducts, the gallbladder, the stomach, and the small and large bowel have to be kept in mind [15,16,17].

In recent years, non-thermal ablative methods have emerged to overcome the limitations of thermal ablations. Irreversible electroporation (IRE) uses electric fields generated between several electrodes to induce irreversible damage to the tumor’s cell membranes [18]. Successful and safe IRE demands careful treatment planning and almost parallel electrode positioning with respect to angulation and skewness in and around the target lesion at defined distances to achieve homogenously high electrical fields [19,20,21]. However, this limits the size of treatable lesions and the access to lesions with complex anatomy [22]. Another non-thermal ablation method is interstitial brachytherapy (ISBT), where locally high radiation doses are applied to treat tumor lesions in the liver [23]. While this method can also be utilized to treat relatively large lesions, the drawback of this treatment option is the lack of repeatability in case of tumor recurrence and that it cannot be used in proximity to radiation-vulnerable adjacent organs and some radio-insensitive tumors.

Electrochemotherapy (ECT) applies the principle of electroporation, as does IRE [24]. This effect does not result in systemic effects and is utilized to achieve a local cytotoxic intracellular concentration of a chemotherapeutic agent. The established chemotherapeutic agents for ECT are bleomycin and cisplatin. Bleomycin is administered either intratumorally or intravenously, whereas cisplatin must be administered directly at the tumor site [25]. Local concentrations of bleomycin are increased by 100–5000 times, and those of cisplatin are increased by 1.8–12.2 times [26]. ECT affects tumor blood vessels through electric pulses alone and drug delivery to the endothelial cells of the tumor vasculature, in addition to its cytotoxic effect on tumor cells [27]. The application of electric pulses essentially causes the tumor vessels to vasoconstrict immediately (vascular lock effect), which is followed by a delayed vascular disrupting effect [28]. It has been demonstrated that this only affects the smaller tumor blood vessels, leaving the larger normal blood vessels around the tumor unaffected. Bleomycin is added to further increase the cumulative effect on tumor blood vessels [29]. Since 1991, ECT has been used to treat cutaneous and subcutaneous metastases of various tumor types. Because of its high rates of local tumor control and good tolerability, it is also included in the current S3 guidelines for the diagnosis and treatment of malignant melanoma [30].

Like IRE, ECT causes only little damage to blood vessels, bile ducts, the liver capsule, and adjacent structures [31,32]. ECT presents potential advantages over IRE in terms of shorter ablation times due to the reduced number of pulses (eight pulses per electrode pair compared to 70–100 pulses per electrode pair), and the ability to place electrodes at a less parallel angle and further apart than IRE (3.0 cm compared to 2.0 cm), thereby enabling the ablation of much larger, geometrically complex tumors in even more anatomically challenging locations without requiring repositioning of the electrodes. Drug-induced lung toxicity and lung fibrosis are possible and feared side effects, commonly appearing at cumulative dosages of 300 mg (300,000 IU) and above [33]. However, if an adult’s body surface area is assumed to be 1.8 m^2^ on average and the suggested bleomycin dose according to the standard operating procedures for an ECT treatment is 15 mg/m^2^, then the 300 mg threshold would not be reached until the twelfth ECT treatment [25]. In addition, the repeatability of ECT is an advantage over ISBT. Technical advances have made ECT available to treat deep-seated tumor lesions in solid organs like the liver. Initial case reports and a pilot study for intraoperative ECT of liver tumors in 16 patients showed promising results with complete ablation rates between 55% and 85% [34,35,36,37]. Percutaneous ECT (pECT) allows a minimally invasive approach to treat deep-seated lesions. A first pilot study with 18 patients, a case series with five patients, and two case reports of pECT for the therapy of liver tumors also showed encouraging results with response rates of 86% to 100% and a primary technique efficacy (PTE is defined as complete ablation of macroscopic tumor following the initial procedure or a defined course of treatment at a prospectively defined time point [38]) of 62% to 80% [39,40,41,42,43,44,45]. However, there is still a lack of evidence concerning the safety and efficacy of ECT for treating liver malignancies, and no literature on long-term follow-up is available.

The incorporation of navigation systems into percutaneous interventions represents a significant advancement in interventional oncology. Navigation systems allow real-time, three-dimensional planning of trajectories and precise targeting of lesions, ensuring accurate electrode placement during ablation procedures and improving PTE [46,47,48,49,50,51]. This technology mitigates the risk of inadvertent damage to surrounding healthy tissues, which leads to fewer complications and thereby enhances the safety profile of ablations and expands their potential applicability in challenging anatomical locations, such as the liver hilum [52,53]. This might significantly benefit complex ablation procedures, where precise placement of multiple electrodes, like in IRE and pECT, is essential for ablation success [54].

Literature on navigation-based treatment planning for ECT is scarce and only available for head and neck tumors [55]. To our knowledge, no study so far has been published focusing on the clinical performance potential of CT-navigated stereotactic percutaneous electrochemotherapy (SpECT) in patients with malignant liver tumors. Because surgery and standard local thermal ablation techniques like MWA and RFA are not always applicable, advanced interventional oncology approaches such as SpECT could be the new silver bullet. Therefore, our retrospective study aimed to report on the safety, efficacy, and outcome of SpECT in patients with primary and secondary liver malignancies.

## 2. Materials and Methods

### 2.1. Patient Slection

The local ethics committee approved this single-arm retrospective cohort study under approval number 24-3930-104. Patient consent was waived due to the retrospective nature of the study. All patient cases were discussed in an interdisciplinary tumor board prior to SpECT, where the recommendation for treatment with SpECT was made because the patients’ lesions were adjacent to central hepatic vessels, central bile ducts, or the hepatic hilum, making resection or thermal ablation unfeasible, and were too large to be treated with IRE. Before the intervention, all patients were provided with comprehensive details and risks of the procedure, and informed consent was obtained. A total of 22 consecutive patients with primary or secondary hepatic malignancies treated with SpECT in a locally curative intention for 23 lesions in 23 sessions between 01/2020 and 05/2024 were included in the study. Exclusion criteria were patient age under 18 years, pregnancy or breastfeeding, and SpECT in palliative intention to reduce tumor volume of a target lesion or in patients with extrahepatic tumor manifestations.

### 2.2. SpECT Procedure and Follow-Up Imaging

All SpECT procedures were carried out under general anesthesia and stereotactic guidance. After the intravenous pressure injection of 100 mL of a non-ionic iodized contrast agent (Accupaque 350, GE Healthcare, Chicago, IL, USA), a dual-phase contrast-enhanced planning CT scan (SOMATOM Definition Edge, Siemens Healthineers AG, Erlangen, Germany) was conducted with an arterial and portal venous phase using sterile radiopaque reflective optical markers that were attached to the patient following sterile preparation. To prevent changes in the liver position brought on by respiratory movement, all CT scans were obtained while the patient was in temporary apnea.

The CT data were then transferred to the navigation system (CAS-One IR, CAScination AG, Bern, Switzerland) adjacent to the CT gantry to define the virtual electrode trajectories using multiplanar reconstructions and three-dimensional models of the scan volume. In detail, trajectory planning was performed by defining a target position (i.e., in and/or around the tumor) and an entry point at skin level by avoiding anatomical risk structures to create an electric field that encompassed the entire tumor volume with a minimum safety margin of 5 mm [56]. The number and arrangement of trajectories were determined by the size and geometry of the target lesions. Subsequently, three to six single-needle electrodes with a diameter of 17 G/1.2 mm and an active tip length of 30 or 40 mm (IGEA VGD, IGEA S.p.A., Carpi, Italy) were introduced through the CAScination aiming device while the patient was in temporary apnea. Before ECT, an unenhanced CT scan was performed. The data of the control scan were transferred to the navigation system and fused with the planning scan based on image coordinates or landmark registration to verify the correct electrode position; if necessary, the electrode position was manually corrected. Bleomycin was administered intravenously through a peripheral venous catheter by hand as a slow bolus over two minutes in a dosage of 15,000 IU/m^2^ body surface area, diluted with saline to 20 mL, followed by a flush of 10 mL saline. After a waiting time of eight minutes to ensure homogenous distribution of the bleomycin in the patient’s body, electroporation was performed using the ECT Pulse Generator IGEA Cliniporator VITAE (IGEA S.p.A., Carpi, Italy) following the standard operating procedures for ECT [25]. Another control scan in the arterial and portal venous phase was performed at the end of the procedure after electrode removal to rule out any peri-interventional complications and to confirm that complete ablation had been achieved. A native control scan was conducted to rule out peri-interventional complications in cases where a second contrast agent injection was not feasible due to impaired renal function. In those cases, a contrast-enhanced MRI and CEUS (contrast-enhanced ultrasound) were conducted on the first postinterventional day to confirm the success of the ablation.

Following SpECT, routine follow-up imaging was carried out at six weeks, three months, and then every three months for two years. The follow-up intervals were then extended to six months. If feasible, an MRI scan was conducted with CareBolus with arterial (after 10 s), late arterial (after 40 s), portal venous (after 75 s), and hepatobiliary late phases (after 20 min), and gadoxetic acid as a contrast agent (Primovist, Bayer AG, Leverkusen, Germany). In rare situations, a CT scan was conducted due to MRI contraindications (for example, implanted pacemakers or allergies to the MRI contrast material).

### 2.3. Data Acquisition and Statistical Analysis

All data were collected retrospectively and consecutively from a database of patients with malignant liver lesions who were treated with CT-guided percutaneous ablation methods at our institution between 01/2020 and 05/2024 and from the patient’s medical reports and electronic charts in our institution’s hospital information system (SAP, Walldorf, Germany). SPSS Version 29 (IBM, New York, NY, USA) was used for all statistical analyses. Patient age, gender, tumor entity, maximum and minimum diameter of the treated lesions, location of the target lesions in the liver, procedure time, the dose length product (DLP) for the procedure, PTE (defined as the percentage of the target tumors that were successfully chemoablated at the 6-week follow-up, as determined by consensus of two experienced radiologists (LL, IE)), local tumor progression (LTP), time to progression (TTP), if applicable, the date and cause of death or duration of follow-up, the occurrence of adverse events according to the CIRSE classification system, and the number of procedure-related side effects (i.e., minimal asymptomatic perihepatic hematoma, asymptomatic pleural effusion, pain, and the post-ablation syndrome) were documented for all patients [38,57].

## 3. Results

### 3.1. Patient Characteristics

From 14 January 2020 to 15 May 2024, 23 tumor lesions in 22 patients were treated in 23 sessions. The mean patient age was 63 years (41 to 80). Seventeen patients were male (77.3%), and five were female (22.7%). Five of the treated HCCs were BCLC stage A, two were BCLC stage B, and one was BCLC stage C. Fourteen of the treated patients had normal liver function parameters at the time of the ablation treatment (63.6%), six had liver cirrhosis Child-Pugh A (27.3%), and two had liver cirrhosis Child-Pugh B (9.1%). Fifteen patients had previously undergone liver surgery and/or MWA, RFA, or IRE for the treatment of liver malignancies (68.2%), eleven patients had received oncologic tumor resections of non-liver-primaries (50.0%), four patients had received trans-arterial chemoembolization (TACE) (18.2%), and twelve patients had received systemic treatment before SpECT (54.5%). Only two patients with HCC were treatment-naive. Table 1 presents a detailed overview of the treated tumor entities and oncological treatments prior to SpECT.

### 3.2. SpECT Procedure, Primary Technique Efficacy, Complications and Side Effects

The mean tumor long axis of the treated lesions was 42 mm (range: 16 mm–72 mm), and the mean tumor short axis was 34 mm (range: 12 mm–55 mm). The mean procedure time for SpECT was 127.0 min (85 min–188 min), and the mean dose length product for the procedures was 2034.7 µGycm (range: 836 µGycm–3388 µGycm).

PTE was achieved in 22 of 23 lesions (95.7%). Only one lesion (metastasis of a small intestine GIST) showed signs of remnant vital tumor tissue at the ablation margin in proximity to the liver hilum in the first follow-up MRI six weeks after treatment; this was confirmed in an additional PET scan, which was ordered following the MRI scan. According to the PET images, the remaining vital metabolic tumor volume was less than 50% of the original tumor before the treatment with SpECT. Unfortunately, the patient also showed several new liver metastases on the first post-therapeutic MRI scan and was, therefore, referred to systemic therapy.

**Figure 1:**
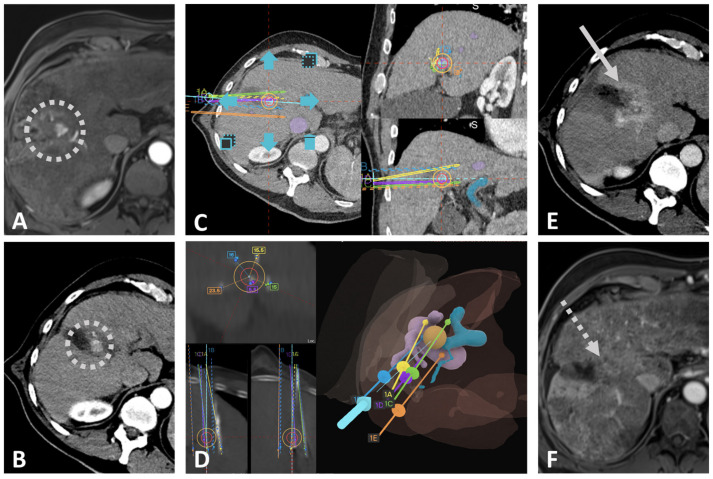
SpECT of a 64-year-old male patient with a 26 mm large iCCA located centrally in Liver Segment IVa/VIII. (**A**) MRI-scan in the arterial phase prior to the SpECT procedure. (**B**) CT-scan in the arterial phase at the start of the SpECT procedure shows the hypervascularized tumor located centrally in liver segments IVa and VIII (dotted circles) with close proximity to the right hepatic artery and the central right bile duct. (**C**) Planning of the trajectories for five ECT-electrodes using multiplanar reconstructions on the stereotactic navigation device, as described before [47,53]. The portal vein and the inferior vena cava including the hepatic veins were segmented in blue and purple, respectively. (**D**) Native control scan fused with the arterial planning scan after stereotactic insertion of the five ECT-electrodes verifies the correct placement of all five electrodes with only minimal deviations of less than two millimeters from the planned trajectories. The 3D model shows the proximity of the target lesion to the portal (blue) and hepatic (purple) veins. (**E**) CT scan in the arterial phase at the end of the SpECT procedure shows complete devascularization of the tumor and the directly adjacent liver tissue with a hyperemic rim around the ablation zone (solid arrow). (**F**) MRI-scan in arterial phase three months after the SpECT procedure shows PTE (dotted arrow indicates the former site of the tumor) without residual vital tumor or LTP.

No major complications (CIRSE grade ≥ 3) were observed. Minor complications and side effects occurred for ten procedures (43.5%) during the hospital stay. Nine of those were minimal perihepatic hematomas in the trajectories of the electrodes, accompanied in 6 (66.7%) by temporary (1–2 days) mild pain at the treated site, which resolved completely without treatment (i.e., side effects, 39.1%). No other side effects (i.e., post-ablation syndrome, pleural effusion) were observed. One patient showed elevated infection parameters without fever or pain the day after the treatment with SpECT. After initiating an antibiotic treatment, the infection parameters returned to normal values, and the patient could be discharged three days after the SpECT treatment (CIRSE grade 2, 4.3%). Figure 1 visualizes the SpECT procedure using the example of an iCCA patient.

### 3.3. Follow-Up, Local Tumor Progression and Time to Progression

The follow-up ranged from 14 days to 39 months (median follow-up 15 months, mean follow-up 14.2 months). No LTP occurred, but six patients (27.3%) had hepatic recurrences at locations unrelated to the lesion treated with SpECT. The mean TTP for those patients was 3.8 months, ranging from two to eight months. One of those patients was treated with MWA for hepatic recurrences two times but developed peritoneal metastases during the follow-up and was thus referred to a systemic treatment. The other five patients with hepatic recurrences also showed extrahepatic tumor progression at the time of diagnosis of the hepatic recurrence. Those patients were either referred to a systemic treatment or the best supportive care.

Nine patients deceased during the follow-up. Notably, one patient died 14 days after the treatment with SpECT from community-acquired pneumonia that was not related to bleomycin exposure. Another patient died 18 days after treatment with SpECT from diffuse mesenteric hemorrhage in the lower abdomen. A possible relation between the treatment with SpECT and the bleeding due to possible deterioration of the patient’s liver function and consecutive coagulopathy was discussed because the patient had an HCC in liver cirrhosis Child-Pugh A with segmental portal vein infiltration. Still, this scenario was ruled unlikely because the last laboratory tests of the patient’s liver function parameters before the hemorrhage showed a normalization of the patient’s liver function to the level measured before the treatment. The other seven patients deceased related to systemic tumor progression or complications related to liver cirrhosis.

## 4. Discussion

To the best of our knowledge, this is the first study investigating the safety, efficacy, and outcome of CT-navigated SpECT in patients with primary and secondary liver malignancies. A PTE of 95.7% without LTP or any major complications approves its outstanding effectiveness and safety profile, highlighting its potential in the growing field of interventional oncology.

Nonthermal ablative modalities are poised to become important components of the interventional oncology armamentarium. Pulsed electric field ablation represents a category of nonthermal therapy that employs the application of electric field pulses to tissue. ECT represents a specific form of pulsed electric field ablation that involves the delivery of electrical energy from electrodes placed around a tumor, resulting in a transient increase in the permeability of the tumor cell membrane. This mechanism allows the intracellular entry of a cytotoxic agent, most commonly bleomycin, which exhibits the significant advantage of being cytotoxic irrespective of the tumor’s histology. ECT is an appealing modality for the treatment of hepatic tumors in sensitive locations due to its capacity to destroy tumor cells while preserving the extracellular matrix of adjacent structures, including major blood vessels and bile ducts [58]. A review of the literature regarding ECT on liver malignancies reveals a paucity of studies and case series examining primarily the intraoperative use of ECT for HCC [36,59], iCCA [60,61], and liver metastases [34,37,62,63]. Of particular note here is a recently published prospective phase II study by Djokic et al. on 24 patients with 32 HCC lesions (mean diameter of 2.5 cm) treated by ECT not amendable for other treatment options [59]. A complete response, partial response, and stable disease were observed in 84.4%, 12.5%, and 3.1% of the treated lesions, respectively, with local tumor control over 50 months of observation in 78.0% of nodules. In addition, no serious adverse events related to the procedure were observed within 24 h postoperatively in the intensive care unit. By comparison, our results show a substantially higher PTE (95.7% vs. 84.4%) and local tumor control rate (100% vs. 78%) with a comparable safety profile, despite a bigger mean long axis tumor diameter of 4.2 cm and our inhomogeneous study population containing various and more aggressive tumors such as iCCA and metastases from colorectal cancer, lung cancer and breast cancer, albeit over a shorter follow-up period with a median of 15 months in our study in comparison to a median of 20 months in Djokic’s study. In another prospective phase II trial regarding colorectal liver metastases (mean diameter 2.0 cm), the objective response rate was 75% (our study: 100%) with a complete and partial response of 63% (our study: 95.7%) and 12% (our study: 4.3%), which is significantly inferior compared to our findings [63]. This clearly emphasizes the potential added value of minimally invasive CT-navigated SpECT for treating primary and secondary liver malignancies. Using sophisticated navigation technology allows us to effectively and safely target malignant liver tumors particularly those located in difficult-to-target intrahepatic locations [46].

To date, only two prior reports with a percutaneous (but not a stereotactic) approach to liver tumors using ECT have been published [42,45]. In the study by Spallek et al. [42], a total of 18 patients with liver tumors (mean long axis tumor diameter of 5.9 cm) of various histopathologic origins (mostly colorectal cancer and breast cancer in 38.9% and 22.2% of cases, respectively; HCC in only 11.1%) were evaluated for safety and efficacy of ECT over time. The objective response rate of 85.7% (complete response 61.9%, partial 23.8%) was substantially lower compared to our results (100%), indicating the potential benefit of using a stereotactic treatment approach. However, the comparatively worse results of Spallek et al. can be at least partially explained by the different entity distribution of tumor lesions and their larger diameter (5.9 cm vs. 4.2 cm). In terms of safety, Spallek et al. reported that 18 out of 21 patients (85.7%) experienced mild or moderate side effects following ECT, without further stratification by the CIRSE classification system, as is the case in this study. According to our results, the overall complication rate of 4.3% is low and in the range of 0% and 57.9% of previously published data on stereotactic liver ablation [46]. Notably, no major complications (CIRSE grade ≥ 3) were observed. In addition, ablation-related side effects were reported in a total of nine treatment sessions (39.1%) in our study. According to Ahmed M et al., side effects are expected, undesired consequences of the procedure that occur frequently but rarely, if ever, resulting in significant morbidity [38]. We think it is important to emphasize the difference between side effects and true complications, as only the latter usually have a relevant impact on further management. To date, the use of different definitions and terminology for safety and complications [38,64,65], respectively, severely limits the comparability between studies, highlighting the critical need for further standardization of adverse event definitions, as noted by Tinguely et al. [46].

In a second study by the same group on local tumor control with different ablation technologies in primary and secondary liver malignancies, 211 lesions were treated in 155 patients, including 21 lesions treated with ECT (mean diameter 6.6 cm) [45]. Similarly to Spallek et al., the most frequent indications for ECT were colorectal cancer metastases in 38%, breast cancer metastases in 24%, and HCC in 14% of treated lesions. Treatment with ECT resulted in a remarkable local tumor control of 81% in 12 months, despite the large size of the treated lesions and their predominant location in challenging sites (91% of cases, i.e., hepatic lesions adjacent to central hepatic vessels, central bile ducts or hepatic hilum). Of note, local tumor control was defined as complete remission, partial remission, or stable disease according to the RECIST version 1.1 criteria [66]. Considering the differences in the heterogeneity of tumor type and extent, the results presented above appear slightly inferior compared to our findings, which observed no local tumor progression in all treated lesions over a mean follow-up of 15 months. Nevertheless, hepatic recurrences at locations unrelated to the treated lesions occurred in six patients (27.3%), with a mean TTP of 3.8 months. To address this problem, the combination of (Sp)ECT and immunotherapy may be a promising approach in the near future to improve local and distant tumor control and enhance the systemic antitumor response [67]. Further studies are warranted to understand the underlying mechanisms and the potential of a combined treatment to enable a more personalized and tailored therapeutic approach.

This study has several limitations. As a single-center retrospective study, the findings may not be fully generalizable to other clinical environments that utilize different equipment, have varying levels of operator experience, or involve diverse patient populations. Additionally, the small sample size and relatively short follow-up period may hinder the robustness of the results. The absence of a control group further complicates the interpretation of treatment efficacy. Moreover, the substantial heterogeneity within the study cohort, which includes patients with different tumor entities, varying histological types, and a range of prior oncological treatments (e.g., liver surgery, systemic treatment, etc.), may significantly limit the ability to detect meaningful variations in treatment outcomes. Finally, while an outcome analysis focusing on overall survival would have been desirable as a primary endpoint, the aforementioned limitations may substantially bias this survival analysis, which is only relevant to the scientific community in strictly defined clinical scenarios. This is exemplified by the CLOCC trial, which demonstrated a significant overall survival benefit of combining local treatment (i.e., RFA with or without surgical resection) plus systemic therapy compared to systemic treatment alone in patients with unresectable colorectal liver metastases without extrahepatic disease [68].

## 5. Conclusions

This study provides the first published evidence that SpECT appears to be a safe and effective new local treatment modality for primary and secondary liver malignancies. SpECT may have a critical impact on clinical management, particularly for tumor lesions that are not amenable to traditional thermoablative procedures due to their size and/or challenging location. The promising impact on outcome in terms of local tumor control may further lead to the establishment of SpECT as an integral part of the minimally invasive armamentarium for the treatment of primary and secondary liver cancer.

## Figures and Tables

**Table 1:** SpECT-treated tumor entities and prior oncological treatments.

Tumor Entity and Pathohistology (G = Grading)	Initial TNM/BCLC	Previous Locoregional Therapy	Previous Systemic Therapy
iCCA, G3	pT2, pN0 (0/5), cM0	Liver resection (segment II–IV, VIII)	Gemcitabine/Cisplatin
iCCA, G3	ypT2, ypN0, cM0	Liver resection (segment V–VIII, atypical II/III)	Gemcitabine/Cisplatin
iCCA, G2-G3	cT1a, cN0, cM0	2x IRE liver (segment VIII)	-
HCC	BCLC B	-	-
HCC, G1	BCLC A	TACE liver (segment III, VI, VIII), RFA liver (segment VI), MWA liver (segment III, IV, VII)	-
HCC, G2	BCLC B	TACE liver (segment II, VII), liver resection (segment IVa, VIII, VII, atypical III resection), MWA liver (segment V, VI/VII, VIII)	-
HCC	BCLC C	TACE/MWA liver (segment IV/V, V)	-
HCC, G1	BCLC A	-	-
HCC, G2	BCLC A	Liver resection (segment IVb, atypical VI), IRE liver (segment IVa)	-
HCC, G2	BCLC A	Liver resection (segment VI, atypical II/III), MWA liver (segment IVb, V)	-
HCC	BCLC A	RFA liver (segment II/III), TACE liver (segment II/III)	-
Metastasis Breast Cancer, G2, Her2neu negative, proliferation rate 15%	pT1c, pN0 (0/6), cM0	Breast resection, liver resection (segment VIII)	Tamoxifen
Metastasis CRC, G3	pT3b, pN1, cM0	Left hemicolectomy, extended left hemihepatectomy (segment II–IV, VIII)	Folfox, 5-FU/FS, Folfiri/Avastin, Capecitabine/Oxaliplatin, Folfiri/Aflibercept
Metastasis CRC	not available	Right hemihepatectomy (segment V–VIII), high anterior rectal resection	Immunochemotherapy
Metastasis CRC, G2	pT3, pN0, cM1	Sigmoid resection, liver resection (segment II, III, atypical V, VI) MWA liver (segment VII)	Panitumumab, FOLFOX6
Metastasis CRC, G2	pT3, pN2b, cM1	Sigmoid resection, liver resection (segment II/III, atypical IVa)	-
Metastasis CRC	T and N unknown, cM1	Rectal resection, liver resection (segment II/III), atypical right lower lung lobe resection	Capecitabine
Metastasis small intestine GIST, Ki67 index 2%	T and N unknown, cM1	Segment resection of the jejunum, multiple resections of the omentum	Imatinib
Metastasis Granulosa Cell Tumor	not available	Sigmoid resection, omentum resection	Caroplatin/Taxol, Topotecan, Caelix, Gemzar, Treosulfan, Doxorubicin/Trabectidin, Cisplatin/Doxorubicin
Metastasis Lung Cancer, adenocarcinoma, EGFR-EXON20-mutation	cT1b, cN2, cM1c	Right upper lobe lung resection	Carboplatin, Nab-Paclitaxel, Pemetrexed, Atezolizumab, Bevazizumab, Mobocertinib
Metastasis Lung Cancer, adenocarcinoma	ypT2b, ypN1 (5/23), M1b	Left upper lobe lung resection	Carboplatin, Pemetrexed, Atezolizumab, Nivolumab/Ipilimumab
Metastasis Malign Melanoma, BRAF wild type	pT2b, pN3c, pM1c	Cheek surgery	Ipilimumab/Nivolumab

## Data Availability

The source data presented in this study are available upon request from the corresponding author. The source data are not publicly available due to patient privacy.

## References

[1] Rumgay H., Arnold M., Ferlay J., Lesi O., Cabasag C.J., Vignat J., Laversanne M., McGlynn K.A., Soerjomataram I. (2022). Global burden of primary liver cancer in 2020 and predictions to 2040. J. Hepatol..

[2] Sung H., Ferlay J., Siegel R.L., Laversanne M., Soerjomataram I., Jemal A., Bray F. (2021). Global Cancer Statistics 2020: GLOBOCAN Estimates of Incidence and Mortality Worldwide for 36 Cancers in 185 Countries. CA Cancer J. Clin..

[3] Horn S.R., Stoltzfus K.C., Lehrer E.J., Dawson L.A., Tchelebi L., Gusani N.J., Sharma N.K., Chen H., Trifiletti D.M., Zaorsky N.G. (2020). Epidemiology of liver metastases. Cancer Epidemiol..

[4] Siegel R.L., Miller K.D., Sauer A.G., Fedewa S.A., Butterly L.F., Anderson J.C., Cercek A., Smith R.A., Jemal A. (2020). Colorectal cancer statistics, 2020. CA Cancer J. Clin..

[5] Riihimäki M., Hemminki A., Fallah M., Thomsen H., Sundquist K., Sundquist J., Hemminki K. (2014). Metastatic sites and survival in lung cancer. Lung Cancer.

[6] Ji L., Cheng L., Zhu X., Gao Y., Fan L., Wang Z. (2021). Risk and prognostic factors of breast cancer with liver metastases. BMC Cancer.

[7] Gandaglia G., Abdollah F., Schiffmann J., Trudeau V., Shariat S.F., Kim S.P., Perrotte P., Montorsi F., Briganti A., Trinh Q. (2014). Distribution of metastatic sites in patients with prostate cancer: A population-based analysis. Prostate.

[8] Hackl C., Neumann P., Gerken M., Loss M., Klinkhammer-Schalke M., Schlitt H.J. (2014). Treatment of colorectal liver metastases in Germany: A ten-year population-based analysis of 5772 cases of primary colorectal adenocarcinoma. BMC Cancer.

[9] Park J., Chen M., Colombo M., Roberts L.R., Schwartz M., Chen P., Kudo M., Johnson P., Wagner S., Orsini L.S. (2015). Global patterns of hepatocellular carcinoma management from diagnosis to death: The BRIDGE Study. Liver Int..

[10] European Association for the Study of the Liver (2018). EASL Clinical Practice Guidelines: Management of hepatocellular carcinoma. J. Hepatol..

[11] Benson A.B., D’angelica M.I., Abbott D.E., Anders R., Are C., Bachini M., Borad M., Brown D., Burgoyne A., Chahal P. (2021). Hepatobiliary Cancers, Version 2.2021, NCCN Clinical Practice Guidelines in Oncology. J. Natl. Compr. Cancer Netw..

[12] Omata M., Cheng A.-L., Kokudo N., Kudo M., Lee J.M., Jia J., Tateishi R., Han K.-H., Chawla Y.K., Shiina S. (2017). Asia–Pacific clinical practice guidelines on the management of hepatocellular carcinoma: A 2017 update. Hepatol. Int..

[13] Van Cutsem E., Cervantes A., Adam R., Sobrero A., van Krieken J.H., Aderka D., Aguilar E.A., Bardelli A., Benson A., Bodoky G. (2016). ESMO consensus guidelines for the management of patients with metastatic colorectal cancer. Ann. Oncol..

[14] Froelich M.F., Schnitzer M.L., Rathmann N., Tollens F., Unterrainer M., Rennebaum S., Seidensticker M., Ricke J., Rübenthaler J., Kunz W.G. (2021). Cost-Effectiveness Analysis of Local Ablation and Surgery for Liver Metastases of Oligometastatic Colorectal Cancer. Cancers.

[15] Bitsch R.G., Düx M., Helmberger T., Lubienski A. (2006). Effects of vascular perfusion on coagulation size in radiofrequency ablation of ex vivo perfused bovine livers. Investig. Radiol..

[16] Pillai K., Akhter J., Chua T.C., Shehata M., Alzahrani N., Al-Alem I., Morris D.L. (2015). Heat sink effect on tumor ablation characteristics as observed in monopolar radiof-requency, bipolar radiofrequency, and microwave, using ex vivo calf liver model. Medicine.

[17] Seror O. (2014). Percutaneous hepatic ablation: What needs to be known in 2014. Diagn. Interv. Imaging.

[18] Davalos R.V., Mir L.M., Rubinsky B. (2005). Tissue ablation with irreversible electroporation. Ann. Biomed. Eng..

[19] Sweeney D.C., Neal R.E., Davalos R.V., Meijerink M.R., Scheffer H.J., Narayanan G. (2017). Multi-scale Biophysical Principles in Clinical Irreversible Electroporation. Irreversible Electroporation in Clinical Practice.

[20] Cindrič H., Miklavčič D., Cornelis F.H., Kos B. (2022). Optimization of Transpedicular Electrode Insertion for Electroporation-Based Treatments of Vertebral Tumors. Cancers.

[21] Cornelis F.H., Cindrič H., Kos B., Fujimori M., Petre E.N., Miklavčič D., Solomon S.B., Srimathveeravalli G. (2020). Peri-tumoral Metallic Implants Reduce the Efficacy of Irreversible Electroporation for the Ablation of Colorectal Liver Metastases. Cardiovasc. Interv. Radiol..

[22] Gupta P., Maralakunte M., Sagar S., Kumar-M P., Bhujade H., Chaluvashetty S.B., Kalra N. (2021). Efficacy and safety of irreversible electroporation for malignant liver tumors: A systematic review and meta-analysis. Eur. Radiol..

[23] Bretschneider T., Peters N., Hass P., Ricke J. (2012). Update interstitielle Brachytherapie. Der Radiol..

[24] Geboers B., Scheffer H.J., Graybill P.M., Ruarus A.H., Nieuwenhuizen S., Puijk R.S., van den Tol P.M., Davalos R.V., Rubinsky B., De Gruijl T.D. (2020). High-Voltage Electrical Pulses in Oncology: Irreversible Electroporation, Electrochemotherapy, Gene Electrotransfer, Electrofusion, and Electroimmunotherapy. Radiology.

[25] Gehl J., Sersa G., Matthiessen L.W., Muir T., Soden D., Occhini A., Quaglino P., Curatolo P., Campana L.G., Kunte C. (2018). Updated standard operating procedures for electrochemotherapy of cutaneous tumours and skin metastases. Acta Oncol..

[26] Miklavčič D., Mali B., Kos B., Heller R., Serša G. (2014). Electrochemotherapy: From the drawing board into medical practice. Biomed. Eng. Online.

[27] Jarm T., Cemazar M., Miklavcic D., Sersa G. (2010). Antivascular effects of electrochemotherapy: Implications in treatment of bleeding metastases. Expert Rev. Anticancer Ther..

[28] Groselj A., Kranjc S., Bosnjak M., Krzan M., Kosjek T., Prevc A., Cemazar M., Sersa G. (2018). Vascularization of the tumours affects the pharmacokinetics of bleomycin and the effectiveness of electrochemotherapy. Basic Clin. Pharmacol. Toxicol..

[29] Markelc B., Sersa G., Cemazar M. (2013). Differential mechanisms associated with vascular disrupting action of electrochemo-therapy: Intravital microscopy on the level of single normal and tumor blood vessels. PLoS ONE.

[30] (2020). S3—Leitlinie zur Diagnostik, Therapie und Nachsorge des Melanoms. J. Dtsch. Dermatol. Ges..

[31] Zmuc J., Gasljevic G., Sersa G., Edhemovic I., Boc N., Seliskar A., Plavec T., Brloznik M., Milevoj N., Brecelj E. (2019). Large Liver Blood Vessels and Bile Ducts Are Not Damaged by Electrochemotherapy with Bleomycin in Pigs. Sci. Rep..

[32] Kos B., Voigt P., Miklavcic D., Moche M. (2015). Careful treatment planning enables safe ablation of liver tumors adjacent to major blood vessels by percutaneous irreversible electroporation (IRE). Radiol. Oncol..

[33] O’sullivan J.M., Huddart R.A., Norman A.R., Nicholls J., Dearnaley D.P., Horwich A. (2003). Predicting the risk of bleomycin lung toxicity in patients with germ-cell tumours. Ann. Oncol..

[34] Edhemovic I., Brecelj E., Gasljevic G., Music M.M., Gorjup V., Mali B., Jarm T., Kos B., Pavliha D., Kuzmanov B.G. (2014). Intraoperative electrochemotherapy of colorectal liver metastases. J. Surg. Oncol..

[35] Gasljevic G., Edhemovic I., Cemazar M., Brecelj E., Gadzijev E.M., Music M.M., Sersa G. (2017). Histopathological findings in colorectal liver metastases after electrochemotherapy. PLoS ONE.

[36] Djokic M., Cemazar M., Popovic P., Kos B., Dezman R., Bosnjak M., Zakelj M.N., Miklavcic D., Potrc S., Stabuc B. (2018). Electrochemotherapy as treatment option for hepatocellular carcinoma, a prospective pilot study. Eur. J. Surg. Oncol. (EJSO).

[37] Coletti L., Battaglia V., De Simone P., Turturici L., Bartolozzi C., Filipponi F. (2017). Safety and feasibility of electrochemotherapy in patients with unresectable colorectal liver metastases: A pilot study. Int. J. Surg..

[38] Ahmed M., Solbiati L., Brace C.L., Breen D.J., Callstrom M.R., Charboneau J.W., Chen M.-H., Choi B.I., De Baère T., Dodd G.D. (2014). Image-guided Tumor Ablation: Standardization of Terminology and Reporting Criteria—A 10-Year Update. Radiology.

[39] Iezzi R., Posa A., Caputo C.T., De Leoni D., Sbaraglia F., Rossi M., Tortora G., Tagliaferri L., Valentini V., Colosimo C. (2023). Safety and Feasibility of Analgosedation for Electrochemotherapy of Liver Lesions. Life.

[40] Djokic M., Dezman R., Cemazar M., Stabuc M., Petric M., Smid L.M., Jansa R., Plesnik B., Bosnjak M., Tratar U.L. (2020). Percutaneous image guided electrochemotherapy of hepatocellular carcinoma: Technological advancement. Radiol. Oncol..

[41] Luerken L., Doppler M., Brunner S.M., Schlitt H.J., Uller W. (2021). Stereotactic Percutaneous Electrochemotherapy as Primary Approach for Unresectable Large HCC at the Hepatic Hilum. Cardiovasc. Interv. Radiol..

[42] Spallek H., Bischoff P., Zhou W., de Terlizzi F., Jakob F., Kovàcs A. (2022). Percutaneous electrochemotherapy in primary and secondary liver malignancies—Local tumor control and impact on overall survival. Radiol. Oncol..

[43] FCornelis F.H., Korenbaum C., Ammar M.B., Tavolaro S., Nouri-Neuville M., Lotz J.P. (2019). Multimodal image-guided electrochemotherapy of unresectable liver metastasis from renal cell cancer. Diagn. Interv. Imaging.

[44] Tarantino L., Busto G., Nasto A., Fristachi R., Cacace L., Talamo M., Accardo C., Bortone S., Gallo P., Tarantino P. (2017). Percutaneous electrochemotherapy in the treatment of portal vein tumor thrombosis at hepatic hilum in patients with hepatocellular carcinoma in cirrhosis: A feasibility study. World J. Gastroenterol..

[45] Kovács A., Bischoff P., Haddad H., Zhou W., Temming S., Schäfer A., Spallek H., Kaupe L., Kovács G., Pinkawa M. (2022). Long-Term Comparative Study on the Local Tumour Control of Different Ablation Technologies in Primary and Secondary Liver Malignancies. J. Pers. Med..

[46] Tinguely P., Paolucci I., Ruiter S.J.S., Weber S., de Jong K.P., Candinas D., Freedman J., Engstrand J. (2021). Stereotactic and Robotic Minimally Invasive Thermal Ablation of Malignant Liver Tumors: A Systematic Review and Meta-Analysis. Front. Oncol..

[47] Beyer L.P., Lürken L., Verloh N., Haimerl M., Michalik K., Schaible J., Stroszczynski C., Wiggermann P. (2018). Stereotactically navigated percutaneous microwave ablation (MWA) compared to conventional MWA: A matched pair analysis. Int. J. Comput. Assist. Radiol. Surg..

[48] Engstrand J., Toporek G., Harbut P., Jonas E., Nilsson H., Freedman J. (2017). Stereotactic CT-Guided Percutaneous Microwave Ablation of Liver Tumors with the Use of High-Frequency Jet Ventilation: An Accuracy and Procedural Safety Study. Am. J. Roentgenol..

[49] Abdullah B.J.J., Yeong C.H., Goh K.L., Yoong B.K., Ho G.F., Yim C.C.W., Kulkarni A. (2015). Robotic-assisted thermal ablation of liver tumours. Eur. Radiol..

[50] Charalampopoulos G., Bale R., Filippiadis D., Odisio B.C., Wood B., Solbiati L. (2023). Navigation and Robotics in Interventional Oncology: Current Status and Future Roadmap. Diagnostics.

[51] Schaible J., Lürken L., Wiggermann P., Verloh N., Einspieler I., Zeman F., Schreyer A.G., Bale R., Stroszczynski C., Beyer L. (2020). Primary efficacy of percutaneous microwave ablation of malignant liver tumors: Comparison of stereotactic and conventional manual guidance. Sci. Rep..

[52] Mbalisike E.C., Vogl T.J., Zangos S., Eichler K., Balakrishnan P., Paul J. (2015). Image-guided microwave thermoablation of hepatic tumours using novel robotic guidance: An early experience. Eur. Radiol..

[53] Lachenmayer A., Tinguely P., Maurer M.H., Frehner L., Knöpfli M., Peterhans M., Weber S., Dufour J., Candinas D., Banz V. (2019). Stereotactic image-guided microwave ablation of hepatocellular carcinoma using a computer-assisted navigation system. Liver Int..

[54] Beyer L.P., Pregler B., Michalik K., Niessen C., Dollinger M., Müller M., Schlitt H.J., Stroszczynski C., Wiggermann P. (2017). Evaluation of a robotic system for irreversible electroporation (IRE) of malignant liver tumors: Initial results. Int. J. Comput. Assist. Radiol. Surg..

[55] Groselj A., Kos B., Cemazar M., Urbancic J., Kragelj G., Bosnjak M., Veberic B., Strojan P., Miklavcic D., Sersa G. (2015). Coupling treatment planning with navigation system: A new technological approach in treatment of head and neck tumors by electrochemotherapy. Biomed. Eng. Online.

[56] Crocetti L., de Baére T., Pereira P.L., Tarantino F.P. (2020). CIRSE Standards of Practice on Thermal Ablation of Liver Tumours. Cardiovasc. Interv. Radiol..

[57] Filippiadis D.K., Binkert C., Pellerin O., Hoffmann R.T., Krajina A., Pereira P.L. (2017). Cirse Quality Assurance Document and Standards for Classification of Complications: The Cirse Classification System. Cardiovasc. Interv. Radiol..

[58] Granata V., Fusco R., D’alessio V., Simonetti I., Grassi F., Silvestro L., Palaia R., Belli A., Patrone R., Piccirillo M. (2023). Percutanous Electrochemotherapy (ECT) in Primary and Secondary Liver Malignancies: A Systematic Review. Diagnostics.

[59] Djokic M., Cemazar M., Bosnjak M., Dezman R., Badovinac D., Miklavcic D., Kos B., Stabuc M., Stabuc B., Jansa R. (2020). A Prospective Phase II Study Evaluating Intraoperative Electrochemotherapy of Hepatocellular Carcinoma. Cancers.

[60] Tarantino L., Busto G., Nasto A., Nasto R.A., Tarantino P., Fristachi R., Cacace L., Bortone S. (2018). Electrochemotherapy of cholangiocellular carcinoma at hepatic hilum: A feasibility study. Eur. J. Surg. Oncol. (EJSO).

[61] Granata V., Palaia R., Albino V., Piccirillo M., Setola S.V., Petrillo A., Izzo F. (2020). Electrochemotherapy of cholangiocellular carcinoma at hepatic hilum: A case report. Eur. Rev. Med. Pharmacol. Sci..

[62] Edhemovic I., Gadzijev E.M., Brecelj E., Miklavcic D., Kos B., Zupanic A., Mali B., Jarm T., Pavliha D., Marcan M. (2011). Electrochemotherapy: A New Technological Approach in Treatment of Metastases in the Liver. Technol. Cancer Res. Treat..

[63] Edhemovic I., Brecelj E., Cemazar M., Boc N., Trotovsek B., Djokic M., Dezman R., Ivanecz A., Potrc S., Bosnjak M. (2020). Intraoperative electrochemotherapy of colorectal liver metastases: A prospective phase II study. Eur. J. Surg. Oncol. (EJSO).

[64] Baerlocher M.O., Nikolic B., Sze D.Y. (2023). Adverse Event Classification: Clarification and Validation of the Society of Interventional Radiology Specialty-Specific System. J. Vasc. Interv. Radiol..

[65] Dindo D., Demartines N., Clavien P.-A. (2004). Classification of Surgical Complications: A new proposal with evaluation in a cohort of 6336 patients and results of a survey. Ann. Surg..

[66] Eisenhauer E.A., Therasse P., Bogaerts J., Schwartz L.H., Sargent D., Ford R., Dancey J., Arbuck S., Gwyther S., Mooney M. (2009). New response evaluation criteria in solid tumours: Revised RECIST guideline (version 1.1). Eur. J. Cancer.

[67] Hadzialjevic B., Omerzel M., Trotovsek B., Cemazar M., Jesenko T., Sersa G., Djokic M. (2023). Electrochemotherapy combined with immunotherapy—A promising potential in the treatment of cancer. Front. Immunol..

[68] Ruers T., Van Coevorden F., Punt C.J.A., Pierie J.-P.E.N., Borel-Rinkes I., Ledermann J.A., Poston G., Bechstein W., Lentz M.-A., Mauer M. (2017). Local Treatment of Unresectable Colorectal Liver Metastases: Results of a Randomized Phase II Trial. JNCI J. Natl. Cancer Inst..

